# Dual Functions of the RFTS Domain of Dnmt1 in Replication-Coupled DNA Methylation and in Protection of the Genome from Aberrant Methylation

**DOI:** 10.1371/journal.pone.0137509

**Published:** 2015-09-18

**Authors:** Ronald Garingalao Garvilles, Takashi Hasegawa, Hironobu Kimura, Jafar Sharif, Masahiro Muto, Haruhiko Koseki, Saori Takahashi, Isao Suetake, Shoji Tajima

**Affiliations:** 1 Laboratory of Epigenetics, Institute for Protein Research, Osaka University, Yamadaoka, Suita, Osaka, 565-0871, Japan; 2 RIKEN Center for Interactive Medical Sciences, 1-7-22, Suehiro-cho, Tsurumi-ku, Yokohama City, Kanagawa, 230-0045, Japan; 3 CREST, JST, Saitama, 332-0012, Japan; CNRS, FRANCE

## Abstract

In mammals, DNA methylation plays important roles in embryogenesis and terminal differentiation *via* regulation of the transcription-competent chromatin state. The methylation patterns are propagated to the next generation during replication by maintenance DNA methyltransferase, Dnmt1, in co-operation with Uhrf1. In the N-terminal regulatory region, Dnmt1 contains proliferating cell nuclear antigen (PCNA)-binding and replication foci targeting sequence (RFTS) domains, which are thought to contribute to maintenance methylation during replication. To determine the contributions of the N-terminal regulatory domains to the DNA methylation during replication, Dnmt1 lacking the RFTS and/or PCNA-binding domains was ectopically expressed in embryonic stem cells, and then the effects were analyzed. Deletion of both the PCNA-binding and RFTS domains did not significantly affect the global DNA methylation level. However, replication-dependent DNA methylation of the differentially methylated regions of three imprinted genes, *Kcnq1ot1/Lit1*, *Peg3*, and *Rasgrf1*, was impaired in cells expressing the Dnmt1 with not the PCNA-binding domain alone but both the PCNA-binding and RFTS domains deleted. Even in the absence of Uhrf1, which is a prerequisite factor for maintenance DNA methylation, Dnmt1 with both the domains deleted apparently maintained the global DNA methylation level, whilst the wild type and the forms containing the RFTS domain could not perform global DNA methylation under the conditions used. This apparent maintenance of the global DNA methylation level by the Dnmt1 lacking the RFTS domain was dependent on its own DNA methylation activity as well as the presence of *de novo*-type DNA methyltransferases. We concluded that the RFTS domain, not the PCNA-binding domain, is solely responsible for the replication-coupled DNA methylation. Furthermore, the RFTS domain acts as a safety lock by protecting the genome from replication-independent DNA methylation.

## Introduction

In vertebrates, the 5^th^ position of cytosine bases in CpG sequences is often methylated. This cytosine methylation contributes to tissue-specific gene expression, silencing of retrotransposons, and, in mammals, genomic imprinting and X-chromosome inactivation. Three DNA methyltransferases have been reported in mammals; two of them, Dnmt3a and Dnmt3b, establish *de novo* methylation patterns [1, 2], and the other one, Dnmt1, maintains DNA methylation patterns during the replication process [3] *via* selective methylation activity towards hemi-methylated DNA [4].

Mouse Dnmt1 comprises 1,620 amino acid residues. The N-terminal 248 amino acid residues form an independent domain (NTD) [5], which binds a variety of factors such as transcription repressor DMAP1 [6], Dnmt3a and Dnmt3b [7], Rb2 [8], PCNA [9], CDKL5 kinase [10], casein kinase 1δ/ε [11], and DNA [5]. From this, it is assumed that the NTD acts as a platform for the factors regulating Dnmt1 through tethering to specific regions. Among these factors, PCNA, which binds DNA polymerases and the factors necessary for replication, and thus is a prerequisite factor for replication, is thought to contribute to maintenance methylation during replication [9]. Dnmt1 deleted the NTD has been solved the crystal structure [12]. Dnmt1 processively methylates hemi-methylated DNA at replication foci *in vivo*. For this processive methylation activity, the PCNA-binding domain (PBD) is dispensable under *in vitro* conditions [4].

According to the three-dimensional structure, the replication foci targeting sequence (RFTS) forms a domain that follows the NTD. Interestingly, the crystal structure of Dnmt1 demonstrated that the RFTS domain is inserted into the catalytic pocket, and its position is stabilized by four hydrogen bonds between the two domains [12]. Because of this, DNA cannot gain access to the catalytic center, and cannot undergo methylation when the substrate DNA is short [13]. This RFTS domain is dispensable for the selective DNA methylation activity towards hemi-methylated DNA *in vitro* [12, 14, 15]. The RFTS domain is reported to be a prerequisite for the tethering of Dnmt1 to the replication region [16]. Recently, it was reported that a point mutation in the Zn-finger motif in the RFTS domain induces global hypomethylation and site-specific hypermethylation, and consequently causes both central and peripheral neurodegeneration with the symptoms of hereditary sensory and autonomic neuropathy with dementia and hearing loss [17]. Mutations that are introduced onto the surface of the RFTS domain in contact with the catalytic domain cause autosomal dominant cerebellar ataxia, deafness and narcolepsy (ADCA-DN) [18]. This may suggest that the proper positioning of the RFTS domain and interaction with the catalytic domain are important for the maintenance DNA methylation.

On the other hand, it was reported that Uhrf1 (Np95) is a prerequisite for replication-dependent DNA methylation in embryonic stem cells (ESC) [19, 20]. The SET and RING-associated (SRA) domain of Uhrf1 selectively binds to hemi-methylated DNA and flips the methylated cytosines out of double-stranded DNA [21–23]. The SRA domain interacts directly with the RFTS domain to allow hemi-methylated DNA access to the catalytic center [13].

In the present study, the contributions of PBD in the NTD and the RFTS domain to the maintenance DNA methylation process in mouse ESC were examined. We found that the NTD containing the PBD was dispensable and that the RFTS domain was necessary for the replication-coupled DNA methylation. Furthermore, the RFTS domain functioned as a safety lock by protecting the genome from replication-independent DNA methylation.

## Materials and Methods

### Mouse Dnmts cDNA

Mouse cDNA coding full-length Dnmt1, oocyte-type Dnmt1, 291–1620, 602–1620 with SV40 nuclear localization signal and myc-tag sequences at their N-termini, and mouse Dnmt3a2 with a TAP tag [24] at the C-terminus were cloned into pCAGIpuro, pCAGIblast, or pCAGIzeo, which were kind gifts from Dr. Niwa (RIKEN Center for Developmental Biology, Kobe, Japan). Each cDNA was constructed by PCR, and all the sequences were confirmed by the dideoxy method [25].

### DNA methylation activity

Recombinants of the full-length Dnmt1, oocyte-type Dnmt1, Dnmt1(291–1620), Dnmt1(602–1620), and full-length Dnmt1 with the H168R mutation were prepared and their DNA methylation activities were determined as described elsewhere [12, 13]. In brief, in a total volume of 25 μl reaction buffer, 6.4 nM Dnmt1, 66 nM DNA, and 2.2 μM [^3^H]-*S*-adenosyl-L-methionine (10 Ci/mmol) (Perkin Elmer) were incubated at 37°C for 1 h, and then the specific activity (mol-CH_3_ transferred to DNA/h/mol Dnmt1 enzyme) was determined within a linear time-course range.

### Mouse ESC

Mouse ESC were cultured in Glasgow Minimum Essential Medium (Sigma Aldrich) supplemented with 15% (v/v) Knockout Serum Replacement (Invitrogen), 1% (w/v) fetal bovine serum (Intergen), 10 units/ml penicillin G, 100 μg/ml streptomycin, 1 mM non-essential amino acids, 100 μM sodium pyruvate, 0.1 mM β-mercaptoethanol, and 0.2% leukemia inhibitory factor, under a 5% CO_2_ atmosphere at 37°C in dishes coated with gelatin. *Dnmt1* or/and *Uhrf1* conditional knockout ESC were prepared from mice embryos as described elsewhere [20, 26]. TKO ESC, which was knocked out *Dnmt1*, *Dnmt3a*, and *Dnmt3b* genes, was kindly provided by Dr. Okano at RIKEN [27].

### Isolation of cells expressing ectopic Dnmt1 or Dnmt3a2

A plasmid harboring Dnmt1 or Dnmt3a2-TAP cDNA (50 μg) was linearized with ScaI, and then transfected into ESC, in which the endogenous *Dnmt1* or/and *Uhrf1* gene was sandwiched between *loxP* sequences or TKO, with a Gene Pulser (BioRad) at 250 V and 500 μF according to the manufacturer’s protocol. After the electroporation, the cells were cultured in the medium supplemented with 1.5 μg/ml puromycin, 20 μg/ml blasticidin S, or 100 μg/ml zeocin, and the resistant clones were isolated. Each clone was analyzed as to the expression of Dnmt1 or Dnmt3a2 by Western blotting (S1 File).

### Deletion of the endogenous *Dnmt1*and *Uhrf1* genes

To delete the endogenous *Dnmt1* or/and *Uhrf1* genes sandwiched with *loxP* sequences, cells were cultured in the presence of 0.8 μM OHT (Sigma Aldrich) [28]. The expression of the endogenous genes was monitored by Western blotting.

### Detection of methylated DNA by dot blotting with anti-methylated cytosine antibody

Genome DNA prepared as described elsewhere [29] was examined as to the DNA methylation level by dot blotting as described [30], except that the DNA was briefly sonicated in a Bioruptor (Cosmo Bio) before alkaline and heat treatment. The DNA blotted onto a nitrocellulose membrane was immuno-detected with anti-methylated cytosine monoclonal antibody FMC-9 (MBL, Japan), followed by incubation with antibodies conjugated with alkaline phosphatase, and then visualized with nitroblue tetrazorium and 5-bromo-4-chloro-3-indolyl-phosphate [31]. The DNA blotted onto a nylon membrane was stained with 0.04% methylene blue in methanol.

### Selective precipitation of methylated DNA fragments with the methylated DNA-binding domain of MBD1

The methylated DNA-binding domain of MBD1 coding 1–75 (MBD1) was used to precipitate methylated DNA fragments [32]. The cDNAs of MBD1 and the point mutant with R22A, which cannot recognize methylated DNA, were subcloned into pET30a with the GST cDNA added at the 5’ end. The recombinant proteins coding (His)_6_-GST-MBD1 and its R22A mutant were purified with Ni-chelate and glutathione-Sepharose columns (GE Healthcare). Methylated genome DNA fragments were selectively precipitated as described elsewhere [33], except for the use of magnet beads, MagneGST (Promega). In brief, DNA was fragmented in a sonicator M220 (Covaris) to obtain an average size of 150 bp [34]. To the fragmented DNA in the buffer comprising 160 mM NaCl, 0.01% (w/v) Tween 20, and 10 mM Tris-HCl, pH 7.5, was added (His)_6_-GST-MBD1(1–75) or its R22A mutant, followed by incubation at 4°C overnight. After the incubation, a 1/10 volume of the mixture was saved for the determination of “input”. The rest of the mixture was washed four times with the same buffer. The input and washed beads were adjusted to 10 mM EDTA, 0.5% (w/v) SDS, and 50 mM Tris-HCl, pH 7.4, and then Proteinase K was added. The mixtures were incubated at 50°C for 3 h. After the digestion reaction, an aliquot of the supernatant fraction was examined in a fluorometer Quantus (Promega) according to the manufacturer’s protocol. The relative level of specific binding was determined by subtracting that on precipitation with the R22A mutant and then normalized as to the input. More than three different preparations of genomic DNA were precipitated, and the averages ± S. D. were determined.

### Bisulfite sequencing

Genome DNA was treated with bisulfite using an EpiTect Bisulfite Kit (Qiagen) with an extended reaction time according to the manufacturer’s protocol, and then amplified with EpiTaq DNA polymerase (Takara) using the following primer sets [35] and conditions. For each of the PCR amplifications, treatment for 2 min at 98°C and 4 min at 72°C was performed initially and after the cycles, respectively.

*IAP gag*: 30 cycles of 98°C for 10 sec, 55°C 30 sec, and 72°C 30 sec with

FW primer: 5’-AAAACACCACAAACCAAAATCTTCTAC-3’

RV primer: 5’-TAAAACATATCCTCTAATCATTTCTACTCA-3’

*Rasgrf1*: 40 cycles of 98°C for 10 sec, 60°C 30 sec, and 72°C 30 sec with

FW primer: 5’-GGAATTTTGGGGATTTTTTAGAGAGTTTATAAAGT-3’

RV primer: 5’-CAAAAACAACAATAATAACAAAAACAAAAACAATAT-3’

*Peg3*: 35 cycles of 98°C for 10 sec, 61°C 30 sec, and 72°C 30 sec with

FW primer: 5’-TTTTGTAGAGGATTTTGATAAGGAGGTGTT-3’

RV primer: 5’-ATCTACAACCTTATCAATTACCCTTAAAAA-3’

*Kcnq1ot1/Lit1*: 30 cycles of 98°C for 10 sec, 58°C 30 sec, and 72°C 60 sec with

FW primer: 5’-ATTTTTGTGGTTTAGGTTTATAGAAGTAGGG-3’

RV primer: 5’-TTAAAAATCACCACAACATAAATAACTATAT-3’

The amplified DNA fragments were subcloned into pBlueScript SKII by TA cloning, and the nucleotide sequences were determined by dideoxy sequencing using the following primers and an ABI Prism 3100 DNA sequencer (Applied Biosystems).

FW primer: 5’-GTTTTCCCAGTCACGACG-3’

RV primer: 5’-GAATTGTGAGCGGATAAC-3’

The results were analyzed with the software described elsewhere [36].

### Immuno-fluorescence observation of ectopically expressed Dnmt1

For ESC expressing Dnmt1 cultured on gelatin-coated cover glasses, the replicating DNA was labeled with a Click-iT EdU Imaging Kit (Molecular Probes). In brief, half of the culture medium was replaced with GMEM containing 2 μM 5-ethynyl-2’deoxyuridine (EdU), followed by incubation at 37°C for 15 min, and then fixing of the cells with 3.7% (v/v) formaldehyde at room temperature for 10 min. The specimens were washed three times, 2 min each, with 125 mM glycine, pH 7.0. The specimens were treated with 0.5% (w/v) Triton X-100 in PBS (PBS-T) at room temperature for 20 min to permeabilize the membranes, and then washed two times, 5 min each, with 125 mM glycine, pH 7.0. The following manipulations were performed in the dark. A Click-iT reaction cocktail containing Alexa Fluor 488 azide was added, followed by incubation at room temperature for 30 min, and then washing three times, 2 min each, with 125 mM glycine, pH 7.0. To this specimen, anti-mouse Dnmt1 antibodies [37] in PBS containing 0.4% (w/v) SDS, 2% (w/v) Triton X-100, and 1% (w/v) bovine serum albumin were added, followed by incubation at 4°C overnight. On the next day, the specimens were washed three times, 5 min each, with PBS-T, and then incubated at room temperature for 1 h with anti-rabbit IgG conjugated with Alexa Fluor 546. After the incubation, the specimens were washed three times with PBS-T, mounted in 50% (v/v) glycerol, and then observed under a laser confocal microscope, Zeiss LSM510.

## Results

### Dnmt1 that lacks the NTD and RFTS domain apparently maintains the global DNA methylation level of the genome

**Fig 1:**
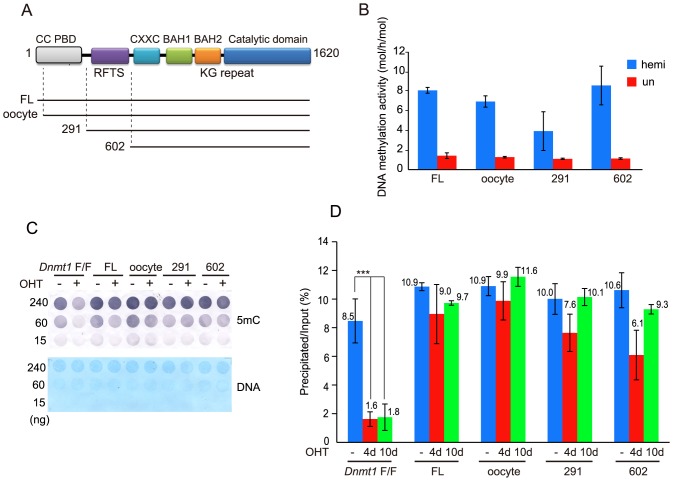
Global DNA methylation in ESC expressing ectopic Dnmt1. (**A**) Schematic illustration of the Dnmt1 structure. Dnmt1 is composed of an N-terminal independently folded domain (NTD) comprising a coiled-coil (CC) and the PBD, the RFTS domain, the CXXC motif, two BAH domains (BAH1 and BAH2), a KG repeat (KG), and a catalytic domain for cytosine methyltransferases. Below the illustration the constructs used in the present study are indicated. (**B**) The DNA methylation activities of full-length Dnmt1 (FL), oocyte-type Dnmt1 (oocyte), Dnmt1(291–1620) (291), and Dnmt1(602–1620) (602). The specific activities (mol/h/mol Dnmt1) towards hemi-methylated (blue, hm) and un-methylated (red, um) DNA are shown as averages ± S. D. (n = 3). (**C**) Genome DNA prepared from ESC expressing no ectopic Dnmt1 (*Dnmt1*-F/F), or full-length Dnmt1 (FL), oocyte-type Dnmt1 (oocyte), Dnmt1(291–1620) (291), or Dnmt1(602–1620) (602) treated without (-) or with OHT (+) for four days was immuno-detected with anti-methylated cytosine antibody (5mC, upper panel) or stained with methylene blue (DNA, lower panel). The amounts of DNA are shown at the left. (**D**) Genome DNA prepared from the ESC treated without (-) or with OHT for four (4d) or ten days (10d) was fragmented by sonication, and then precipitated with MBD1. The precipitated DNA was normalized as to that of input DNA. The precipitated DNA is expressed as the averages ± S. D. for more than three independently prepared genome preparations for before and after the OHT treatment, respectively. All the ESC expressing ectopic Dnmt1 showed significant methylation levels compared to that without ectopic Dnmt1 after the OHT treatment (p<0.001) except for the ESC expressing Dnmt1(602–1620), which was p<0.01. **, p<0.01, and *** p<0.001.

Full-length Dnmt1 possesses multi-domains, which comprise the NTD containing the PBD [5], the RFTS, the CXXC motif, two BAH domains, and the catalytic domain (Fig 1A). The catalytic domain with the CXXC motif and the two BAH domains are reported to selectively methylate hemi-methylated DNA, which is a prerequisite property for maintenance DNA methylation [12, 14, 38]. Recombinant Dnmt1s coding the full-length, oocyte-type [39], NTD-deleted {Dnmt1(291–1620)}, and both NTD and RFTS domain-deleted {Dnmt1(602–1620)} forms exhibited similar selectivity and specific activities towards hemi-methylated DNA (Fig 1B). On the other hand, the PBD and RFTS domains are reported to be necessary for targeting replication foci for the maintenance DNA methylation in culture cells [9, 16]. These reports, however, did not confirm whether or not the PBD and/or RFTS domain are necessary for the maintenance DNA methylation in cells.

To examine the roles of the PBD and RFTS domain, stable clones expressing the full-length Dnmt1, oocyte-type Dnmt1, Dnmt1(291–1620), and Dnmt1(602–1620) with a myc-tag at their N-termini in mouse ESC, in which the endogenous *Dnmt1* gene in the genome is sandwiched between *loxP* sequences (*Dnmt1*-F/F), were established. Clones expressing ectopic Dnmt1 at a similar to or lower level than that of endogenous Dnmt1 were established (S1 Fig), and then the endogenous *Dnmt1*-F/F was removed by 4-hydroxytamoxifen (OHT) treatment. Four days, not two days, treatment was sufficient to completely remove the endogenous *Dnmt1* (S2 Fig). The DNA methylation level of the genome DNA from the cells cultured for four days was determined by dot blotting (Fig 1C). The DNA prepared from the parent cells four days after the treatment with OHT showed a significantly reduced DNA methylation level due to global DNA demethylation. On the other hand, the genome DNA prepared from all the clones expressing ectopic Dnmt1 showed maintenance of the DNA methylation level, indicating that the NTD and RFTS domain are apparently dispensable for maintenance of the global genome methylation level in ESC. This was further confirmed by the precipitation method with the methylated DNA-binding domain of MBD1 (MBD1) (Fig 1D) [33]. Genome DNA of ESC ectopically expressing either of the constructs was significantly precipitated with the MBD1 even after ten days cultivation in the presence of OHT, indicating that the Dnmt1 construct with the NTD and RFTS domain deleted apparently possesses the ability to maintain the global DNA methylation level.

**Fig 2:**
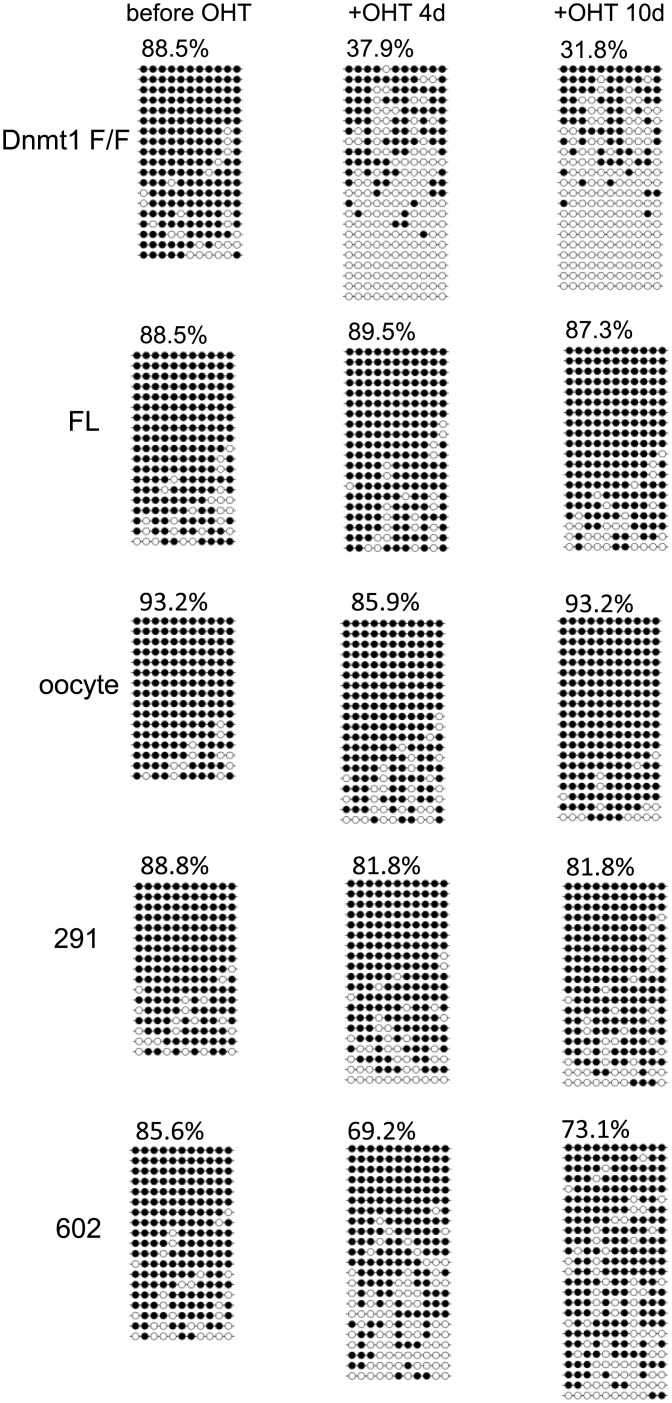
DNA methylation analyses of the *gag* of *IAP*. The methylation state of the genome DNA prepared from ESC expressing no Dnmt1 (*Dnmt1* F/F), or full-length Dnmt1 (FL), oocyte-type Dnmt1 (oocyte), Dnmt1(291–1620) (291), or Dnmt1(602–1620) (602) four or ten days after addition of OHT. The methylation states of the *gag* of *IAP* are shown with that of the parent cells before (*Dnmt1*-F/F) and after the OHT treatment (*Dnmt1*-F/F+OHT). Each horizontal line indicates the CpGs in one analyzed clone. Each circle indicates one CpG site, methylated (filled circles) or un-methylated (open circles). The percentages of methylation are indicated at the top.

In general, retrotransposons in the genome are densely methylated and thus silenced. For one of the retrotransposons, intracisternal A particle (IAP), the methylation level was determined (Fig 2). The CpG sites in IAP *gag* were 88.5% methylated in the parent ESC, the level being reduced to 37.9 and 31.8% after four and ten days treatment with OHT to delete the endogenous *Dnmt1*-F/F (*Dnmt1*-F/F+OHT). In ESC ectopically expressing full-length Dnmt1, oocyte-type Dnmt1, Dnmt1(291–1620), and Dnmt1(602–1620), the DNA methylation levels after 10-days treatment with OHT at the IAP-*gag* were 87.3, 93.2, 81.8 and 73.1%, respectively. This again supports that the deletion mutants apparently possess the ability to maintain the global genome methylation level.

### The RFTS is necessary for Dnmt1 to be localized to the replication foci

**Fig 3:**
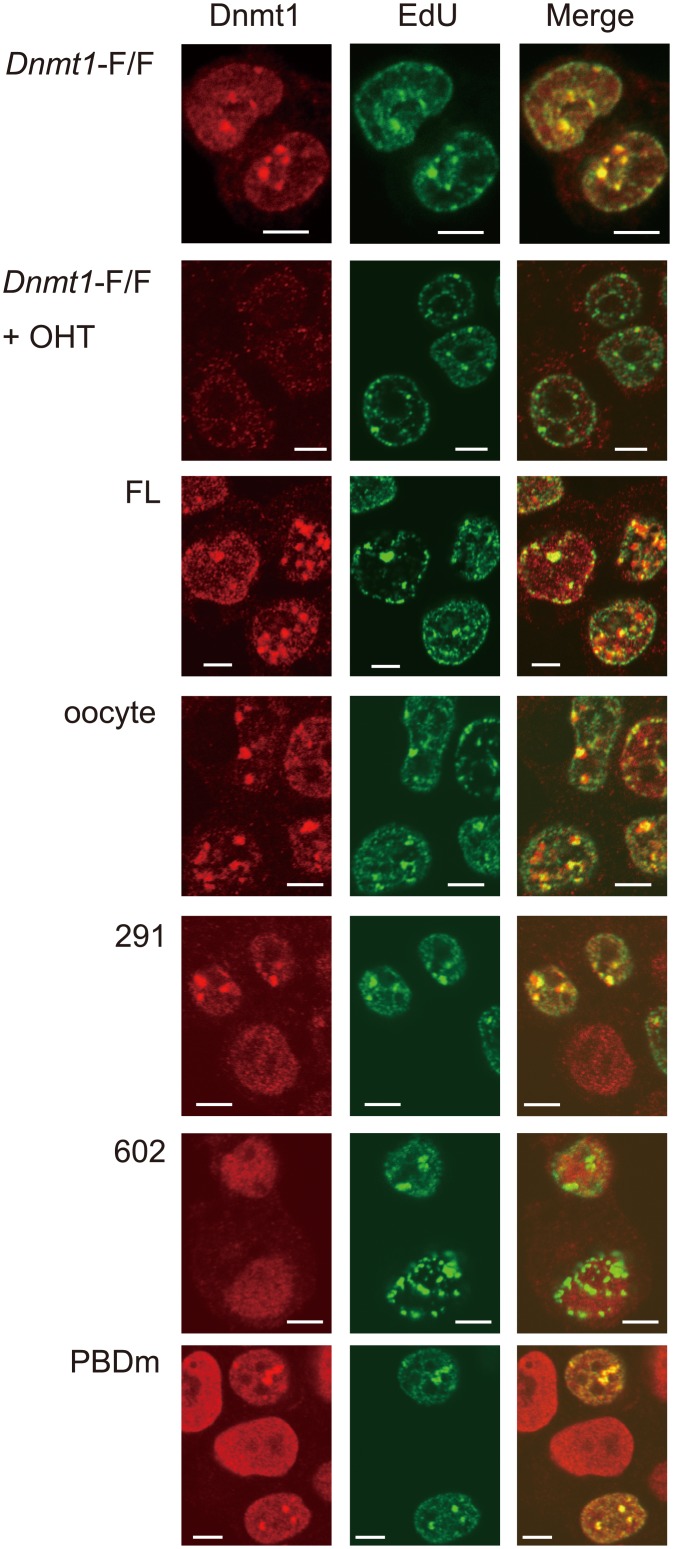
Localization of Dnmt1 at the replication region in ESC. ESC (*Dnmt1*-F/F), and ESC expressing no Dnmt1 (*Dnmt1*-F/F +OHT), or ectopic full-length Dnmt1 (FL), oocyte-type Dnmt1 (oocyte), Dnmt1(291–1620) (291), Dnmt1(602–1620) (602), or full-length Dnmt1 with the mutation of H168R (PBDm) treated with OHT were labeled with EdU, and detected the EdU (green) and Dnmt1 (red), and the images were merged. White bars indicate 5 μm.

As it was reported that the RFTS domain is a prerequisite for Dnmt1 to be localized to the replication region [16], and that PCNA, which is known to be a platform for the replication machinery, also contributes to the localization of Dnmt1 to the replication region [9], we did not expect that the N-terminal region, which contains the PBD and RFTS domain, was dispensable for maintaining the global DNA methylation level. Therefore, we examined the localization of the Dnmt1s used in the present study at replication foci (Fig 3). Expectedly, Dnmt1(602–1620) was not co-localized with EdU, which is incorporated at the replication time and thus accumulates at replication foci. All the other Dnmt1s containing the RFTS domain were co-localized with EdU. This is consistent with the previous report that the RFTS is necessary for Dnmt1 to be localized at replication foci [16].

This raised the questions whether or not the apparent retention of global methylation by ectopically expressed Dnmt1(602–1620) was the result of replication-coupled DNA methylation, and whether or not the PBD contributes to the maintenance methylation during replication.

### The RFTS domain, not the PBD, is necessary for replication-coupled DNA methylation

To examine replication-coupled DNA methylation, we monitored the differentially methylated regions (DMR) of imprinted genes. Imprinted genes show mono-allelic expression due to complete or null DNA methylation at the DMR in male- or female-germ cell-derived loci. The methylation patterns of DMR of imprinted genes cannot be restored once the methylation patterns are erased [40, 41]. Therefore, the DMR of an imprinted gene is an ideal indicator for evaluating the replication-coupled DNA methylation by ectopically expressed Dnmt1 after deletion of the endogenous *Dnmt1*-F/F.

**Fig 4:**
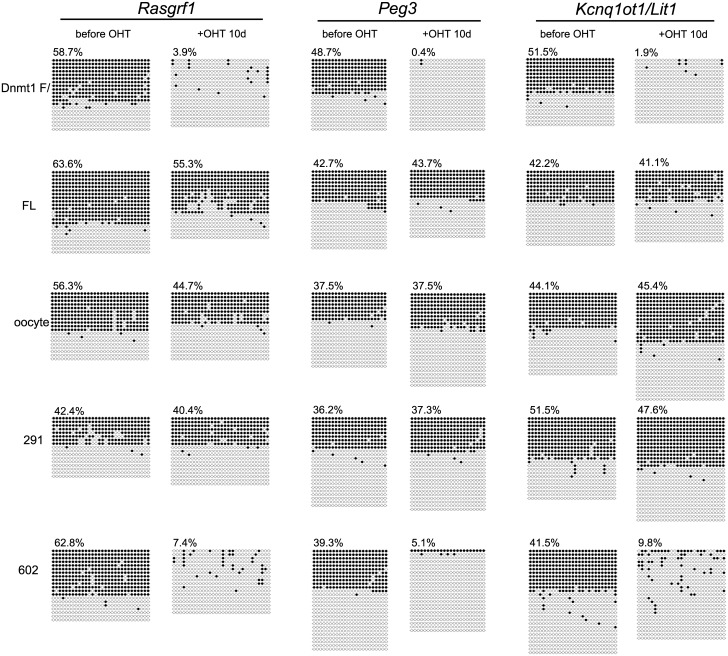
DNA methylation analyses of the DMR of three imprinted genes. The methylation states of the DMRs of *Rasgrf1*, *Peg3*, and *Kcnq1ot1/Lit* are shown with that of the parent cells before and after ten days OHT treatment (+OHT 10d). Each horizontal line indicates the CpGs in one analyzed clone. Each circle indicates one CpG site, methylated (filled circles) or un-methylated (open circles). The percentages of methylation are indicated at the top.

For the DMRs of three imprinted genes, *Rasgrf1* [42], *Peg3* [43], and *Kcnq1ot1/Lit1* [44], the methylation state was determined (Fig 4). Expectedly, ESC expressing the full-length Dnmt1 or oocyte-type Dnmt1, which is specifically expressed in oocytes and at an early stage of embryogenesis, maintained the DMR methylation judging from that about half of the subcloned DMR of *Rasgrf1*, *Peg3*, and *Kcnq1ot1/Lit1* exhibited almost full methylation after deletion of the endogenous *Dnmt1* gene, which is an indication of mono-allelic DMR methylation. Dnmt1(291–1620), which lacks the PBD but contains the RFTS domain, maintained the full methylation of DMR in about half the clones tested for all three imprinted genes. On the contrary, with Dnmt1(602–1620), which lacks both the PBD and RFTS domain, DNA methylation in the DMR was lost.

**Fig 5:**
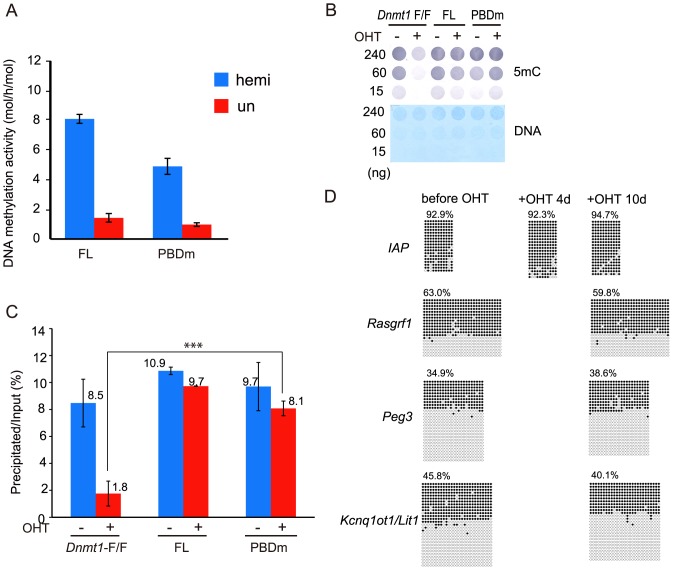
DNA methylation of ESC expressing full-length Dnmt1 with a mutation in the PBD. (**A**) DNA methylation activity of the recombinant full-length Dnmt1 (FL) and that with the H168R mutation (PBDm) was determined. The specific activities (mol/h/mol Dnmt1) towards hemi-methylated (hm) and un-methylated (um) DNA are shown as averages ± S. D. (n = 3). (**B**) Genome DNA prepared from the cells before and after deletion of the endogenous *Dnmt1* gene with OHT for ten days was immuno-blotted with anti-methylated cytosine antibody (5mC, upper panel) or stained with methylene blue (DNA, lower panel). The amounts of DNA are shown at the left. (**C**) Genome DNA prepared as in B was sonicated and precipitated with MBD1, and then quantitated, averages ± S. D. (n = 3) being shown as in Fig 1D. The values for the parent genome before and after the OHT-treatment were taken from Fig 1D. ***, P<0.001. (**D**) Genome DNA prepared as in B was analyzed as to the methylation state at the *IAP* and DMR of three imprinted genes, *Rasgrf1*, *Peg3*, and *Kcnq1ot1/Lit*, in PBDm cells as in Figs 2 and 4. The percentages of methylation are indicated at the top.

The results clearly indicate that the PBD is dispensable for the replication-coupled DNA methylation at the DMR in imprinted genes. To further test this, full-length Dnmt1 with a mutation of H168R in the PBD, this mutation being reported to be localized to the replication region [45] but not to interact with PCNA [46], was introduced into *Dnmt1*-F/F ESC. The DNA methylation activity of the recombinant Dnmt1 with the mutation (PBDm) was similar to that of the wild-type Dnmt1 (Fig 5A). Ten days after addition of OHT to delete the endogenous *Dnmt1*-F/F, the global DNA methylation level was determined by dot blotting with anti-methylated cytosine antibody and precipitation assaying with MBD1. The DNA methylation level did not show any difference from that of the parent ESC or expressed wild-type Dnmt1 (Fig 5B and 5C). Furthermore, the apparent DNA methylation level at IAP and the DNA methylation patterns at the DMR of imprinted genes in the cells expressing the mutant Dnmt1 were maintained (Fig 5D). In addition, as reported, Dnmt1 with the H168R mutation was localized to the replication region (Fig 3). These results indicate that Dnmt1 binding to PCNA is dispensable for maintenance DNA methylation. It can also be concluded that the RFTS is a prerequisite domain for the replication-coupled DNA methylation *in vivo*.

### The RFTS domain protects the genome from replication-independent DNA methylation

Dnmt1(602–1620) lacking the RFTS domain expressed in ESC apparently could maintain the global DNA methylation level, however, it was not co-localized to the replicating region (Fig 3) [16], and could not maintain the DMR methylation in the imprinted genes (Fig 4). It was reported that Uhrf1, which binds specifically to hemi-methylated DNA that appears just after the replication, is a prerequisite factor for the DNA methylation during replication [20], and directly interacts with the RFTS to remove it from the catalytic pocket [13]. Judging from these findings, the apparent maintenance of the global DNA methylation level by Dnmt1(602–1620) may not co-operate with Uhrf1.

**Fig 6:**
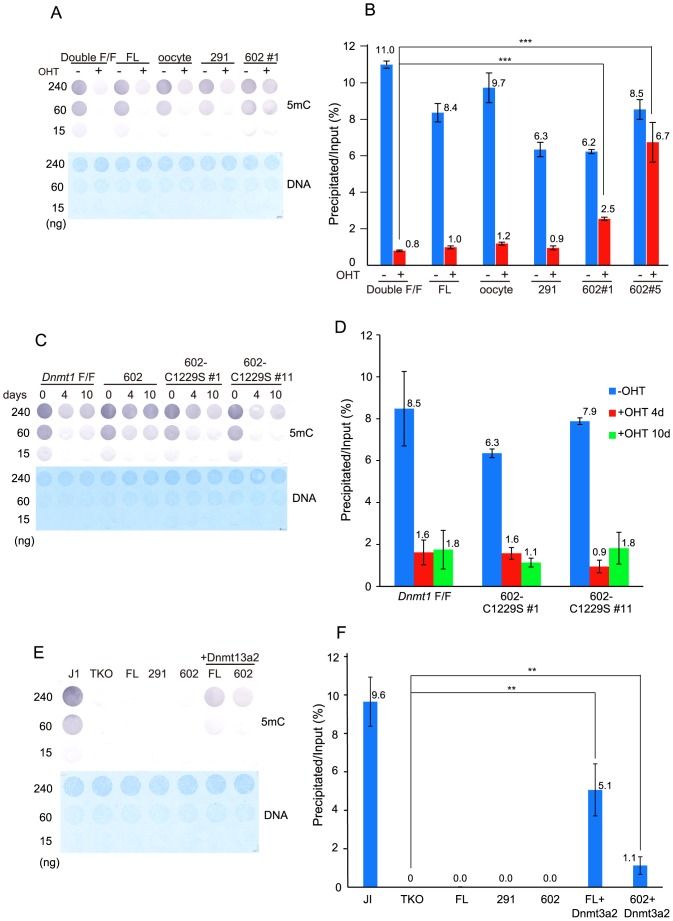
Global DNA methylation of *Dnmt1* and *Uhrf1* double-knockout, or TKO ESC expressing ectopic Dnmt1. (**A**) Genome DNA prepared from *Dnmt1* and *Uhrf1* conditional double-knockout ESC expressing either no ectopic Dnmt1 (Double F/F), or full-length Dnmt1 (FL), oocyte-type Dnmt1 (oocyte), Dnmt1(291–1620) (291), or Dnmt1(602–1620) clone #1 (602#1) treated without (-) or with OHT (+) for ten days was immuno-blotted with anti-methylated cytosine antibody (5mC, upper panel) or stained with methylene blue (DNA, lower panel). The amounts of DNA are shown at the left. (**B**) The genome DNA in panel A and Dnmt1(602–1620) clone #5 (602#5) were fragmented by sonication, and then precipitated with MBD1. The precipitated DNA was quantitated as in Fig 1D, averages ± S. D. being shown. ***, p<0.001. (**C**) Genome DNA prepared from *Dnmt1* conditional-knockout ESC expressing either no ectopic Dnmt1 (*Dnmt1*-F/F), Dnmt1(602–1620), Dnmt1(602–1620) with C1229S clones#1 (602-C1229S #1), or clone #11 (602-C1229S #11) treated without (0) or with OHT for four days (4) or ten days (10) was immuno-blotted with anti-methylated cytosine antibody (5mC, upper panel), or stained with methylene blue (DNA, lower panel). The amounts of DNA are shown at the left. (**D**) Genome DNA prepared from *Dnmt1* conditional-knockout ESC expressing either no ectopic Dnmt1 (*Dnmt1*-F/F), or Dnmt1(602–1620) with C1229S clone #1 (602-C1229S #1) or clone #11 (602-C1229S #11) treated without (blue) or with OHT for four days (red) or ten days (green) was fragmented by sonication, and then precipitated with MBD1. The precipitated DNA was quantitated as in Fig 1D, averages ± S. D. being shown. (**E**) Genome DNA prepared from parent ESC (J1), TKO ESC (TKO), TKO ESC expressing full-length Dnmt1 (FL), Dnmt1(291–1620) (291), Dnmt1(602–1620) (602), full-length Dnmt1 and Dnmt3a2-TAP (FL+Dnmt3a2), or (602–1620) and Dnmt3a2-TAP (602+Dnmt3a2) was immuno-blotted with anti-methylated cytosine antibody (5mC, upper panel), or stained with methylene blue (DNA, lower panel). The amounts of DNA are shown at the left. (**F**) Genome DNA prepared from J1, TKO ESC (TKO), TKO ESC expressing no ectopic Dnmt1 (TKO), or full-length Dnmt1 (FL), Dnmt1(291–1620) (291), Dnmt1(602–1620) (602), full-length Dnmt1 and Dnmt3a2-TAP (FL+Dnmt3a2), or Dnmt1(602–1620) and Dnmt3a2-TAP (602+Dnmt3a2) was fragmented by sonication, precipitated with MBD1, and then analyzed as in Fig 1D. The value obtained for TKO was considered as no DNA methylation and subtracted from each measurement. Averages ± S. D. are shown.

To determine whether or not Dnmt1(602–1620) co-operates with Uhrf1, the expression plasmids of Dnmt1 were transfected into ESC with *Dnmt1* and *Uhrf1* double conditional knockout (Double F/F), and the endogenous *Dnmt1*-F/F and *Uhrf1*-F/F were deleted after isolation of the clones ectopically expressing Dnmt1. For the clones expressing ectopic Dnmt1(602–1620) (S1 Fig), the global methylation levels showed a significant level of DNA methylation (Fig 6A and 6B), the DNA methylation level being significantly higher than in ESC with Double F/F or expressing the full-length Dnmt1, oocyte-type Dnmt1, or Dnmt1(291–1620) after deletion of the endogenous *Dnmt1* and *Uhrf1* genes with OHT. This finding indicates that Dnmt1(602–1620) lacking the RFTS domain may methylate the genome independent of Uhrf1, that is, the RFTS domain inserted into the catalytic pocket of Dnmt1(291–1620) acts as a fail-safe mechanism ensuring replication-coupled DNA methylation in the genome. Since this global methylation by Dnmt1(602–1620) was not observed for its catalytic activity dead mutant (Fig 6C and 6D), in which the Cys residue at 1229 was replaced with Ser [45], the DNA methylation activity of Dnmt1(602–1620) was necessary for the apparent global methylation.

### *De novo* methylation activity is necessary for the replication-independent DNA methylation by Dnmt1 that lacks the NTD and RFTS domain

As for the replication-independent methylation by Dnmt1(602–1620) that lacks the NTD and RFTS domain, two possibilities should be considered; Dnmt1(602–1620) itself creates and maintains global methylation, or fully methylates the hemi-methylated CpG produced by *de novo* methyltransferases, Dnmt3a2 and/or Dnmt3b. To examine this, Dnmt1(602–1620) was expressed in the *Dnmt1*, *Dnmt3a*, and *Dnmt3b* triple knockout (TKO) ESC, and then the global genome methylation levels were determined. Identical to the TKO cells expressing full-length Dnmt1 or Dnmt1(291–1620), the DNA methylation in the cells expressing Dnmt1(602–1620) was below the detection level on anti-methylated cytosine antibody reactivity and MBD1 precipitation analysis (Fig 6E and 6F). Furthermore, the expression of TAP-tagged Dnmt3a2 in the cells expressing Dnmt1(602–1620) led to significant recovery of the global DNA methylation level. These results indicate that the aberrant DNA methylation observed in Double F/F ESC expressing Dnmt1(602–1620) was due to the replication-independent DNA methylation by Dnmt1(602–1620) at the hemi-methylated CpG sites, which may have been created by *de novo*-type DNA methyltransferse Dnmt3a2 or Dnmt3b.

## Discussion

In the present study, the contribution of the NTD containing the PBD and the RFTS domain to the DNA methylation in ESC was examined. It was found that the RFTS domain, not the PBD, is necessary for the replication-coupled DNA methylation. Importantly, the RFTS domain also protects the genome DNA from replication-independent DNA methylation.

### The RFTS domain is necessary for replication-coupled DNA methylation

The recombinant Dnmt1(602–1620), which lacks the NTD and RFTS domain, selectively methylates hemi-methylated DNA, and its specific activity is similar to that of the full-length, oocyte-type, or Dnmt1(291–1620) (Fig 1B). However, because the RFTS domain is responsible for the tethering of Dnmt1 to the replication region (ref. 16; see Fig 3), Dnmt1(602–1620) could not perform replication-coupled DNA methylation at the DMR of imprinted genes, this process being dependent on the existence of Uhrf1 at replication foci [20]. Recently, it was reported that the SRA domain of Uhrf1 interacts directly with the RFTS domain [13, 47], which plugs the catalytic pocket of Dnmt1, to remove the domain from the catalytic pocket [13].

It was reported that the 255–291 and 191–324 sequences of Dnmt1, which code the sequences between the NTD and the RFTS domain [12], and part of the NTD [5], respectively, are necessary for the maintenance methylation in ESC [41]. This observation is distinct from our present results. Because the 1–248 sequence of the NTD is protease-resistant [5], and the 291–356 sequence in the crystal structure of Dnmt1(291–1620) could not be deduced [12], possibly due to its flexible structure, deletion of the 255–291 and 191–324 sequences may change the relative positions of the NTD and RFTS domain and/or destroy the three-dimensional structure of either domain. This could be the reason for the lack of recovery of the maintenance methylation with the reported deletion constructs. Different from in the study by Borowczyk et al. [41], the constructs used in the present study were able to form an active structure *in vitro*, and thus the requirement solely of the RFTS domain for replication-coupled methylation in ESC is conclusive.

### The PBD is not necessary for maintenance DNA methylation

PCNA, encircling DNA binds DNA polymerase δ and many factors contributing to replication, is a prerequisite factor for replication [48]. Dnmt1 binds PCNA through the PBD in the NTD [9]. In addition, the N-terminal domain containing the PCNA binding domain is suggested to contribute to maintenance of DNA methylation [49]. Recently, it was proposed that the interaction of PCNA with Dnmt1 facilitates tethering of Dnmt1 to replication sites to ensure propagation of DNA methylation [50]. However, the present study indicates that the NTD containing the PBD is dispensable for both the maintenance of global DNA methylation patterns and those of imprinted genes in ESC (Figs 1C, 1D and 4). The difference between our present results and those reported by others may be partly due to the expression level of the ectopically expressed Dnmt1. We conclude that the interaction between PCNA and Dnmt1 is not necessarily required for the replication-coupled DNA methylation.

### The RFTS domain protects the genome from replication-independent DNA methylation

Uhrf1 is a necessary factor for replication-coupled methylation, as the *Uhrf1*-knockout ESC exhibited a decreased global methylation level [20] (see also Fig 6A and 6B). From this, it was expected that the ectopically expressed Dnmt1 constructs could not maintain DNA methylation in the *Dnmt1* and *Uhrf1* double-knockout ESC. However, Dnmt1(602–1620), which lacks the RFTS domain, showed a significant level of global methylation (Fig 6A and 6B). This is clearly an indication that the methylation of the genome by Dnmt1(602–1620) does not need the co-operation of Uhrf1. That is to say, the RFTS domain protects the genome from replication-independent DNA methylation by plugging the catalytic pocket. The masking of the catalytic pocket by the RFTS domain is important for preventing the access of DNA to the catalytic center [12, 51]. Hemi-methylated DNA is passed onto Dnmt1 through interaction of the RFTS domain with Uhrf1 at the replication region only during replication [13]. The RFTS domain’s position and interaction with Uhrf1 govern the precise timing and location of the maintenance methylation. Anchoring of the RFTS domain in the catalytic pocket is stabilized by four hydrogen bonds with the catalytic domain [12]. Recently, patients with autosomal dominant cerebellar ataxia, deafness and narcolepsy due to point mutations at the interacting surface between the RFTS and catalytic domains were reported [18]. In addition, it was also reported that the RFTS-deleted Dnmt1 enhances tumorigenicity [52]. The mutations may affect the protective role of the RFTS domain by disturbing the proper insertion of the domain into the catalytic pocket.

### *De novo* methylation activity is necessary for fixing the replication-independent DNA methylation

ESC with the *Dnmt1* gene deleted still expressed *de novo*-type DNA methyltransferase Dnmt3a2, which is an isoform lacking the N-terminal 218 residues, and Dnmt3b at high levels [53, 54]. This suggests that the genome is continuously undergoing *de novo* methylation. Nevertheless, methylation on the genome was abolished after four-day culture in *Dnmt1*-knocked out ESC (see Fig 1C and 1D). The methylation marks formed by Dnmt3a2 and Dnmt3b are reported to be specifically hydroxylated through Tet activity and to be removed during replication because hemi-hydroxymethyl CpG is not a good substrate for the maintenance methylation by Dnmt1 and Uhrf1 [55]. Judging from this, most of the *de novo* sites methylated by Dnmt3a2 and Dnmt3b are not fixed on the genome even in the presence of Dnmt1.

On the other hand, the ectopic expression of Dnmt1(602–1620), which lacks the NTD and RFTS domain, apparently maintained the global methylation level of the genome only when Dnmt3a2 and/or Dnmt3b were expressed (Fig 6A, 6B, 6E and 6F). It was strongly suggested that Dnmt1(602–1620) may fix *de novo* methylated sites, which are created by *de novo*-type DNA methyltransferases Dnmt3a2 and Dnmt3b, by fully methylating the hemi-methylated sites, as Dnmt1(602–1620) still possesses selective methylation activity towards hemi-methylated DNA (see Fig 1B). It is thus rational to speculate that Dnmt1(602–1620), which lacks the NTD and RFTS domain, may establish and maintain the DNA methylation introduced by Dnmt3a2 and/or Dnmt3b at any time throughout the cell cycle.

The global DNA methylation level was much higher for the ESC expressing full-length Dnmt1 and Dnmt3a2 than those expressing Dnmt1(602–1620) and Dnmt3a2. Since the NTD is reported to bind Dnmt3a and Dnmt3b [7], in the present study, it is possible that full-length Dnmt1 efficiently coupled with the *de novo* DNA methylation activity of Dnmt3a2 in a replication-coupled manner.

## Conclusions

The RFTS domain is reported to be a prerequisite for the tethering of Dnmt1 to the replication region for DNA methylation. The crystal structure of Dnmt1 demonstrated that the RFTS domain is inserted into the catalytic pocket and its position is stabilized by four hydrogen bonds between the two domains. In the present study we have shown that the RFTS domain is necessary not only for the replication-coupled DNA methylation in mouse ESC, but also that it function as a safety lock by protecting the genome from replication-independent DNA methylation. Furthermore, the interaction between PCNA and Dnmt1 is not necessarily required for the replication-coupled DNA methylation.

## Supporting Information

S1 Fig Western blotting of ectopically expressed Dnmt1. (**A**) Cell extracts of parent *Dnmt1*-F/F ESC, and cells ectopically expressing full-length Dnmt1 (FL), oocyte-type Dnmt1 (oocyte), Dnmt1(291–1620) (291) clones #4 and 5, or Dnmt1(602–1620) (602), before (-) and after (+) OHT-treatment, were electrophoresed in SDS-polyacrylamide gels, and then Dnmt1 and tubulin were detected by Western blotting. (**B**) Cell extracts of parent *Dnmt1*-F/F ESC, and cells ectopically expressing full-length Dnmt1, and the mutant with H168R (PBDm) were electrophoresed and immuno-detected as in panel A. (**C**) Cell extracts of parent *Dnmt1*-F/F and *Uhrf1*-F/F ESC (Double F/F), and Double F/F ESC ectopically expressing full-length Dnmt1 (FL), oocyte-type Dnmt1 (oocyte), Dnmt1(291–1620) (291), or Dnmt1(602–1620) (602) clone #1 or 5, before (-) and after (+) OHT-treatment, were electrophoresed and immuno-detected as in panel A. (**D**) Cell extracts of parent *Dnmt1*-F/F, and cells ectopically expressing Dnmt1(602–1620) (602) with C1229S #1 (602-C1229S #1) or C1229S #11 (602-C1229S #11), before (-) and after (+) OHT-treatment, were electrophoresed and immuno-detected as in panel A. (**E**) Cell extracts of parent ESC (J1), TKO cells, and TKO cells ectopically expressing full-length Dnmt1 (FL), Dnmt1(291–1620) (291), Dnmt1(602–1620) (602), full-length Dnmt1 and Dnmt3a2-TAP (FL+Dnmt3a2), and Dnmt1(602–1620) and Dnmt3a2-TAP (602+Dnmt3a2) were electrophoresed, and Dnmt1 and Dnmt3a were immuno-detected. Asterisks indicate ectopically expressed Dnmt1 or Dnmt3a2TAP. Molecular size markers are shown at the left of the gel.
(PDF)

S2 Fig Determination of the deletion of the endogenous *Dnmt1* gene by PCR amplification. (**A**) Schematic illustration of the *loxP* inserted sites of the *Dnmt1* gene, and the positions of the primers **a** and **b** (arrows). The numbers in boxes are exon numbers. To determine *Dnmt1* deletion before and after OHT treatment, primers **a** and **b**, as follows were used. The amplification reaction comprised a cycle of denaturation at 94°C 2 min, and then 30 cycles of denaturation at 94°C 30 sec, annealing at 60°C for 30 sec, and extension at 72°C 1 min. Primer **a**: 5’-GTAAGTCTGTCCTTTTTCCCAGTTT-3’, and Primer **b**: 5’- AAACCAGTATGTCTCGTGTCCTTAC-3’. Successful deletion of the endogenous *Dnmt1* diminishes amplification of the 351 bp + loxP (34 bp) size fragment with the (**a** + **b**) primer set. (**B**) PCR amplification of the endogenous *Dnmt1* gene of *Dnmt1*-F/F cells, and cells ectopically expressing full-length Dnmt1 (FL), oocyte-type Dnmt1 (oocyte), Dnmt1(291–1620) (291), and Dnmt1(602–1620) (602#1), before (-) and after (+) OHT treatment. PCR amplifications were performed after two (upper panel) and four days (lower panel) treatment with OHT. Four days, not two days, treatment of the ESC with OHT completely deleted the endogenous *Dnmt1* gene. Molecular size markers (M) are indicated at the right side of the gels.
(PDF)

S1 File Western blotting. (DOCX)

## References

[1] Okano M, Xie S, Li E. Cloning and characterization of a family of novel mammalian DNA (cytosine-5) methyltransferases. Nat Genet. 1998; 19: 219–220. 9662389 10.1038/890

[2] Aoki A, Suetake I, Miyagawa J, Fujio T, Chijiwa T, Sasaki H, et al Enzymatic properties of de novo-type mouse DNA (cytosine-5) methyltransferases. Nucl Acids Res. 2001; 29: 3506–3512. 11522819 10.1093/nar/29.17.3506 PMC55888

[3] Goll MG, Bestor TH. Eukaryotic cytosine methyltransferases. Annu Rev Biochem. 2005; 74: 481–514. 15952895 10.1146/annurev.biochem.74.010904.153721

[4] Vilkaitis G, Suetake I, Klimasauskas S, Tajima S. Processive methylation of hemimethylated CpG sites by mouse Dnmt1 DNA methyltransferase. J Biol Chem. 2005; 280: 63–72. 10.1074/jbc.M411126200 15509558

[5] Suetake I, Hayata D, Tajima S. The amino-terminus of mouse DNA methyltransferase 1 forms an independent domain and binds to DNA with the sequence involving PCNA binding motif. J Biochem. 2006; 140: 763–776. 17046852 10.1093/jb/mvj210

[6] Rountree MR, Bachman KE, Baylin SB. DNMT1 binds HDAC2 and a new co-repressor, DMAP1, to form a complex at replication foci. Nat Genet. 2000; 25: 269–277. 10888872 10.1038/77023

[7] Kim GD, Ni J, Kelesoglu N, Roberts RJ, Pradhan S. Co-operation and communication between the human maintenance and de novo DNA (cytosine-5) methyltransferases. EMBO J. 2002; 21: 4183–4195. 12145218 10.1093/emboj/cdf401 PMC126147

[8] Pradhan S, Kim GD. The retinoblastoma gene product interacts with maintenance human DNA (cytosine-5) methyltransferase and modulates its activity. EMBO J. 2002; 21: 779–788. 11847125 10.1093/emboj/21.4.779 PMC125847

[9] Chuang L, Ian HI, Koh TW, Ng HH, Xu G, Li BF. Human DNA-(Cytosine-5) methyltransferase-PCNA complex as a target for p21WAF1. Science. 1997; 277: 1996–2000. 9302295 10.1126/science.277.5334.1996

[10] Kameshita I, Sekiguchi M, Hamasaki D, Sugiyama Y, Hatano N, Suetake I, et al Cyclin-dependent kinase-like 5 binds and phosphorylates DNA methyltransferase 1. Biochem Biophys Res Commun. 2008; 377: 1162–1167. 10.1016/j.bbrc.2008.10.113 18977197

[11] Sugiyama Y, Hatano N, Sueyoshi N, Suetake I, Tajima S, Kinoshita E, et al The DNA-binding activity of mouse DNA methyltransferase 1 is regulated by phosphorylation with casein kinase 1δ/ε. Biochem J. 2010; 427: 489–497. 10.1042/BJ20091856 20192920

[12] Takeshita K, Suetake I, Yamashita E, Suga M, Narita H, Nakagawa A, et al Structural insight into maintenance methylation by mouse DNA methyltransferase 1 (Dnmt1). Proc Natl Acad Sci USA. 2011; 108: 9055–9059. 10.1073/pnas.1019629108 21518897 PMC3107267

[13] Berkyurek AC, Suetake I, Arita K, Takeshita K, Nakagawa A, Shirakawa M, et al The DNA methyltransferase Dnmt1 directly interacts with the SET and RING finger associated (SRA) domain of the multifunctional protein Uhrf1 to facilitate accession of the catalytic center to hemi-methylated DNA. J Biol Chem. 2014; 289: 379–386. 10.1074/jbc.M113.523209 24253042 PMC3879560

[14] Song J, Rechkoblit O, Bestor TH, Patel DJ. Structure of DNMT1-DNA complex reveals a role for autoinhibition in maintenance DNA methylation. Science. 2011; 331: 1036–1040. 10.1126/science.1195380 21163962 PMC4689315

[15] Song J, Teplova M, Ishibe-Murakami S, Patel DJ. Structure-based mechanistic insights into DNMT1-mediated maintenance methylation. Science. 2012; 335: 709–712. 10.1126/science.1214453 22323818 PMC4693633

[16] Leonhardt H, Page AW, Weier HU, Bestor TH. A targeting sequence directs DNA methyltransferase to sites of DNA replication in mammalian nuclei. Cell. 1992; 71: 865–873. 1423634 10.1016/0092-8674(92)90561-p

[17] Klein CJ, Botuyan MV, Wu Y, Ward CJ, Nicholson GA, Hammans S, et al Mutations in DNMT1 cause hereditary sensory neuropathy with dementia and hearing loss. Nat Genet. 2011; 43: 595–600. 10.1038/ng.830 21532572 PMC3102765

[18] Winkelmann J, Lin L, Schormair B, Kornum BR, Faraco J, Plazzi G, et al Mutations in DNMT1 cause autosomal dominant cerebellar ataxia, deafness and narcolepsy. Hum Mol Genet. 2012; 21: 2205–2210. 10.1093/hmg/dds035 22328086 PMC3465691

[19] Bostick M, Kim JK, Estève PO, Clark A, Pradhan S, Jacobsen SE. UHRF1 plays a role in maintaining DNA methylation in mammalian cells. Science. 2007; 317: 1760–1764. 17673620 10.1126/science.1147939

[20] Sharif J, Muto M, Takebayashi S, Suetake I, Iwamatsu A, Endo TA, et al The SRA protein Np95 mediates epigenetic inheritance by recruiting Dnmt1 to methylated DNA. Nature. 2007; 450: 908–913. 17994007 10.1038/nature06397

[21] Arita K, Ariyoshi M, Tochio H, Nakamura Y, Shirakawa M. Recognition of hemi-methylated DNA by the SRA protein UHRF1 by a base flipping mechanism. Nature. 2008; 455: 818–822. 10.1038/nature07249 18772891

[22] Awakumov GV, Walker JR, Xue S, Li Y, Duan S, Bronner C, et al Structural basis for recognition of hemi-methylated DNA by the SRA domain of human UHRF1. Nature. 2008; 455: 822–826. 10.1038/nature07273 18772889

[23] Hashimoto H, Horton JR, Zhang X, Bostick M, Jacobsen SE, Cheng X. The SRA domain of UHRF1 flips 5-methylcytosine out of the DNA helix. Nature. 2008; 455: 826–830. 10.1038/nature07280 18772888 PMC2602803

[24] Puig O, Caspary F, Rigaut G, Rutz B, Bouveret E, Bragado-Nilsson E, et al The tandem affinity purification (TAP) method: A general procedure of protein complex purification. Methods. 2001; 24: 218–229. 11403571 10.1006/meth.2001.1183

[25] Sanger F, Nicklen S, Coulson AR. DNA sequencing with chain-terminating inhibitors. Proc Natl Acad Sci USA. 1977; 74: 5463–5467. 271968 10.1073/pnas.74.12.5463 PMC431765

[26] Jackson-Grusby L, Beard C, Possemato R, Tudor M, Fambrough D, Csankovszki G, et al Loss of genomic methylation causes p53-dependent apoptosis and epigenetic deregulation. Nat Genet. 2001; 27: 31–39. 11137995 10.1038/83730

[27] Tsumura A, Hayakawa T, Kumaki Y, Takebayashi S, Sakaue M, Matsuoka C, et al Maintenance of self-renewal ability of mouse embryonic stem cells in the absence of DNA methyltransferases Dnmt1, Dnmt3a and Dnmt3b. Genes Cells. 2006; 11: 805–814. 16824199 10.1111/j.1365-2443.2006.00984.x

[28] Matsui T, Leung D, Miyashita H, Maksakova IA, Miyachi H, Kimura H, et al Proviral silencing in embryonic stem cells requires the histone methyltransferase ESET. Nature. 2010; 464: 927–931. 10.1038/nature08858 20164836

[29] Sambrook J, Russell DW. Molecular Cloning, A Laboratory Manual. 3rd edition New York: Cold Spring Harbor Laboratory Press; 2001.

[30] Brown T. Dot and slot blotting of DNA. Curr Protoc Mol Biol. 2001 May Chapter2: Unit2. 9B. 10.1002/0471142727.mb0209bs21. 10.1002/0471142727.mb0209bs21 18265189

[31] Harland RM. *In situ* hybridization: an improved whole-mount method for *Xenopus* embryos. Methods Cell Biol. 1991; 36: 685–695. 1811161 10.1016/s0091-679x(08)60307-6

[32] Ohki I, Shimotake N, Fujita N, Jee J, Ikegami T, Nakao M, et al Solution structure of the methyl-CpG binding domain of human MBD1 in complex with methylated DNA. Cell. 2001; 105: 487–497. 11371345 10.1016/s0092-8674(01)00324-5

[33] Morita S, Takahashi RU, Yamashita R, Toyoda A, Horii T, Kimura M, et al Genome-wide analysis of DNA methylation and expression of microRNA in breast cancer cells. Int J Mol Sci. 2012; 13: 8259–8272. 10.3390/ijms13078259 22942701 PMC3430232

[34] Taiwo O, Wilson GA, Morris T, Seisenberger S, Reik W, Pearce D, et al Methylome analysis using MeDIP-seq with low DNA concentrations. Nat Protoc. 2012; 7: 617–636. 10.1038/nprot.2012.012 22402632

[35] Tomizawa S, Kobayashi H, Watanabe T, Andrews S, Hata K, Kelsey G, et al Dynamic stage-specific changes in imprinted differentially methylated regions during early mammalian development and prevalence of non-CpG methylation in oocytes. Development. 2011; 138: 811–820. 10.1242/dev.061416 21247965 PMC3035086

[36] Kumaki Y, Oda M, Okano M. QUMA. Quantification tool for methylation analysis. Nucl Acids Res. 2008; 36: W170–175. 10.1093/nar/gkn294 18487274 PMC2447804

[37] Takagi H, Tajima S, Asano A. Overexpression of DNA methyltransferase in myoblast cells accelerates myotube formation. Eur J Biochem. 1995; 231: 282–291. 7635139 10.1111/j.1432-1033.1995.tb20698.x

[38] Bashtrykov P, Jankevicius G, Smarandache A, Jurkowska RZ, Ragozin S, Jeltsch A. Specificity of Dnmt1 for methylation of hemimethylated CpG sites resides in its catalytic domain. Chem Biol. 2012; 19: 572–578. 10.1016/j.chembiol.2012.03.010 22633409

[39] Mertineit C, Yoder JA, Taketo T, Laird DW, Trasler JM, Bestor TH. Sex-specific exons control DNA methyltransferase in mammalian germ cells. Development. 1998; 125: 889–897. 9449671 10.1242/dev.125.5.889

[40] Tucker KL, Beard C, Dausmann J, Jackson-Grusby L, Laird PW, Lei H, et al Germ-line passage is required for establishment of methylation and expression patterns of imprinted but not of nonimprinted genes. Genes Dev. 1996; 10: 1008–1020. 8608936 10.1101/gad.10.8.1008

[41] Borowczyk E, Mohan KN, D'Aiuto L, Cirio MC, Chaillet JR. Identification of a region of the DNMT1 methyltransferase that regulates the maintenance of genomic imprints. Proc Natl Acad Sci USA. 2009; 106: 20806–20811. 10.1073/pnas.0905668106 19923434 PMC2791569

[42] Plass C, Shibata H, Kalcheva I, Mullins L, Kotelevtseva N, Mullins J, et al Identification of Grf1 on mouse chromosome 9 as an imprinted gene by RLGS-M. Nat Genet. 1996; 14: 106–109. 8782830 10.1038/ng0996-106

[43] Kuroiwa Y, Kaneko-Ishino T, Kagitani F, Kohda T, Li LL, Tada M, et al Peg3 imprinted gene on proximal chromosome 7 encodes for a zinc finger protein. Nat Genet. 1996; 12: 186–190. 8563758 10.1038/ng0296-186

[44] Engemann S, Strödicke M, Paulsen M, Franck O, Reinhardt R, Lane N, et al Sequence and functional comparison in the Beckwith-Wiedemann region: implications for a novel imprinting centre and extended imprinting. Hum Mol Genet. 2000; 9: 2691–2706. 11063728 10.1093/hmg/9.18.2691

[45] Takebayashi S, Tamura T, Matsuoka C, Okano M. Major and essential role for the DNA methylation mark in mouse embryogenesis and stable association of DNMT1 with newly replicated regions. Mol Cell Biol. 2007; 27: 8243–8258. 17893328 10.1128/MCB.00899-07 PMC2169176

[46] Iida T, Suetake I, Tajima S, Morioka H, Ohta S, Obuse C, et al PCNA clamp facilitates action of DNA cytosine methyltransferase 1 on hemimethylated DNA. Genes Cells. 2002; 7: 997–1007. 12354094 10.1046/j.1365-2443.2002.00584.x

[47] Bashtrykov P, Jankevicius G, Jurkowska RZ, Ragozin S, Jeltsch A. The Uhrf1 protein stimulates the activity and specificity of the maintenance DNA methyltransferase Dnmt1 by an allosteric mechanism. J Biol Chem. 2014; 289: 4106–4115. 10.1074/jbc.M113.528893 24368767 PMC3924276

[48] Moldovan GL, Pfander B, Jentsch S. PCNA, the maestro of the replication fork. Cell. 2007; 129: 665–679. 17512402 10.1016/j.cell.2007.05.003

[49] Egger G, Jeong S, Escobar SG, Cortez CC, Li TW, Saito Y, et al Identification of DNMT1 (DNA methyltransferase 1) hypomorphs in somatic knockouts suggests an essential role for DNMT1 in cell survival. Proc Natl Acad Sci USA. 2006; 103: 14080–14085. 16963560 10.1073/pnas.0604602103 PMC1599915

[50] Schneider K, Fuchs C, Dobay A, Rottach A, Qin W, Wolf P, et al Dissection of cell cycle—dependent dynamics of Dnmt1 by FRAP and diffusion-modeling. Nucl Acids Res. 2013; 41: 4860–4876. 10.1093/nar/gkt191 23535145 PMC3643600

[51] Bashtrykov P, Rajavelu A, Hackner B, Ragozin S, Carell T, Jeltsch A. Targeted mutagenesis results in an activation of DNA methyltransferase 1 and confirms an autoinhibitory role of its RFTS domain. Chembiochem. 2014; 15: 743–748. 10.1002/cbic.201300740 24532244

[52] Wu BK, Mei SC, Brenner C. RFTS-deleted DNMT1 enhances tumorigenicity with focal hypermethylation and global hypomethylation. Cell Cycle. 2014; 13: 3222–3231. 10.4161/15384101.2014.950886 25485502 PMC4615144

[53] Chen T, Ueda Y, Xie S, Li E. A novel Dnmt3a isoform produced from an alternative promoter localizes to euchromatin and its expression correlates with active de novo methylation. J Biol Chem. 2002; 277: 38746–38754. 12138111 10.1074/jbc.M205312200

[54] Watanabe D, Suetake I, Tada T, Tajima S. Stage- and cell-specific expression of Dnmt3a and Dnmt3b during embryogenesis. Mech Dev. 2002; 118: 187–190. 12351185 10.1016/s0925-4773(02)00242-3

[55] Otani J, Kimura H, Sharif J, Endo TA, Mishima Y, Kawakami T, et al Cell cycle-dependent turnover of 5-hydroxymethyl cytosine in mouse embryonic stem cells. PLoS One. 2013; 8: e82961 10.1371/journal.pone.0082961 24340069 PMC3858372

