# Supplementary figures and images for: Dual Functions of the RFTS Domain of Dnmt1 in Replication-Coupled DNA Methylation and in Protection of the Genome from Aberrant Methylation
Source: PLoS One. 2015 Sep 18;10(9):e0137509. doi: 10.1371/journal.pone.0137509 (PMC4575159; doi:10.1371/journal.pone.0137509)

SI Figure S1. Western blotting of ectopically expressed Dnmt1

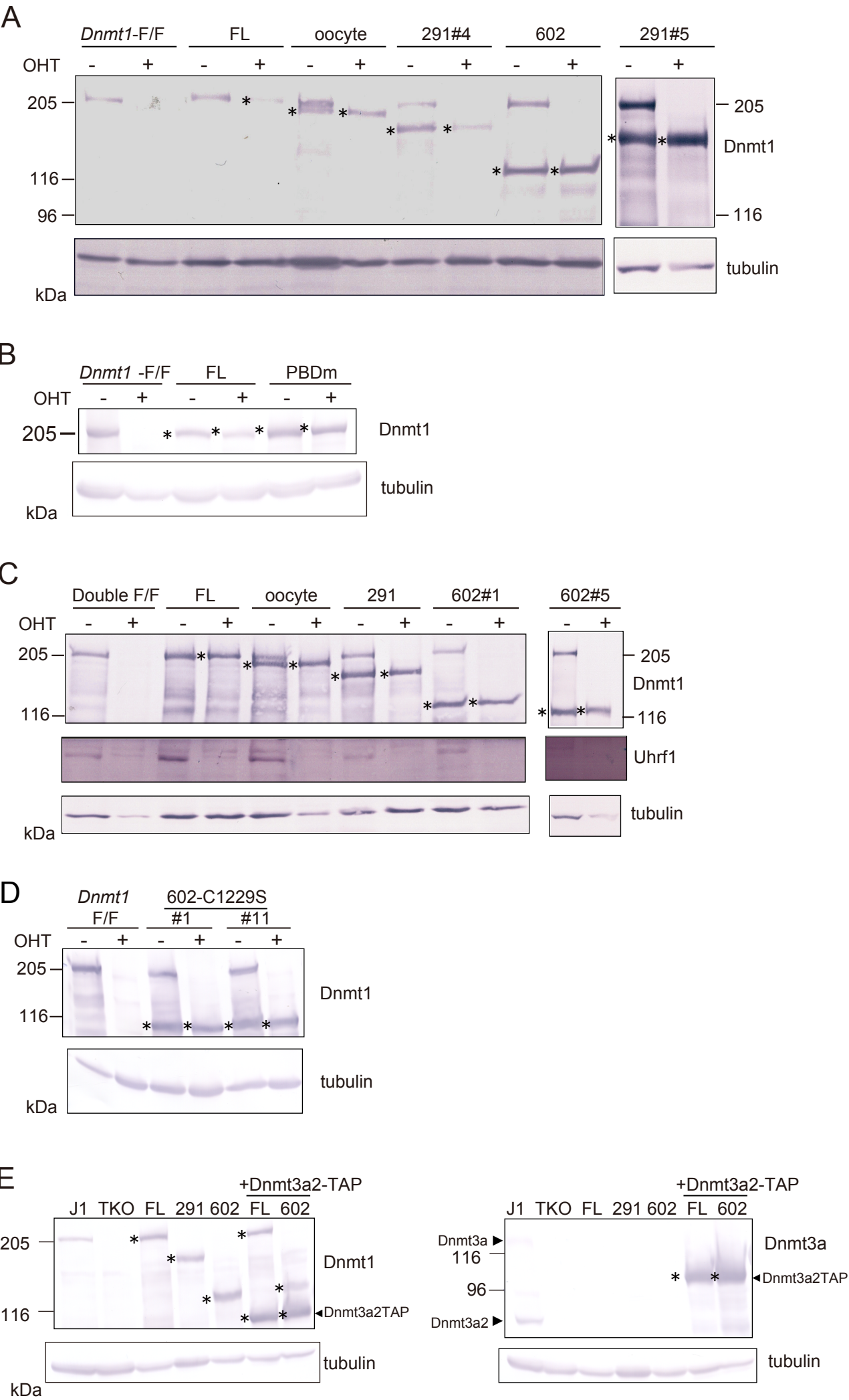

Supplement: S1 Fig — (A) Cell extracts of parent Dnmt1-F/F ESC, and cells ectopically expressing full-length Dnmt1 (FL), oocyte-type Dnmt1 (oocyte), Dnmt1(291–1620) (291) clones #4 and 5, or Dnmt1(602–1620) (602), before (-) and after (+) OHT-treatment, were electrophoresed in SDS-polyacrylamide gels, and then Dnmt1 and tubulin were detected by Western blotting. (B) Cell extracts of parent Dnmt1-F/F ESC, and cells ectopically expressing full-length Dnmt1, and the mutant with H168R (PBDm) were electrophoresed and immuno-detected as in panel A. (C) Cell extracts of parent Dnmt1-F/F and Uhrf1-F/F ESC (Double F/F), and Double F/F ESC ectopically expressing full-length Dnmt1 (FL), oocyte-type Dnmt1 (oocyte), Dnmt1(291–1620) (291), or Dnmt1(602–1620) (602) clone #1 or 5, before (-) and after (+) OHT-treatment, were electrophoresed and immuno-detected as in panel A. (D) Cell extracts of parent Dnmt1-F/F, and cells ectopically expressing Dnmt1(602–1620) (602) with C1229S #1 (602-C1229S #1) or C1229S #11 (602-C1229S #11), before (-) and after (+) OHT-treatment, were electrophoresed and immuno-detected as in panel A. (E) Cell extracts of parent ESC (J1), TKO cells, and TKO cells ectopically expressing full-length Dnmt1 (FL), Dnmt1(291–1620) (291), Dnmt1(602–1620) (602), full-length Dnmt1 and Dnmt3a2-TAP (FL+Dnmt3a2), and Dnmt1(602–1620) and Dnmt3a2-TAP (602+Dnmt3a2) were electrophoresed, and Dnmt1 and Dnmt3a were immuno-detected. Asterisks indicate ectopically expressed Dnmt1 or Dnmt3a2TAP. Molecular size markers are shown at the left of the gel. (PDF) [file pone.0137509.s001.pdf]

SI Figure S2. Determination of the deletion of endogenous Dnmt1 by PCR

A

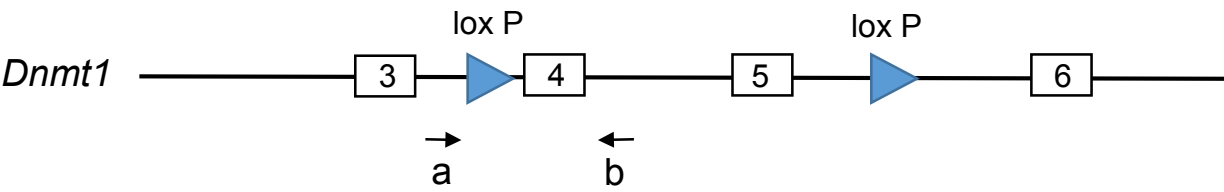

B

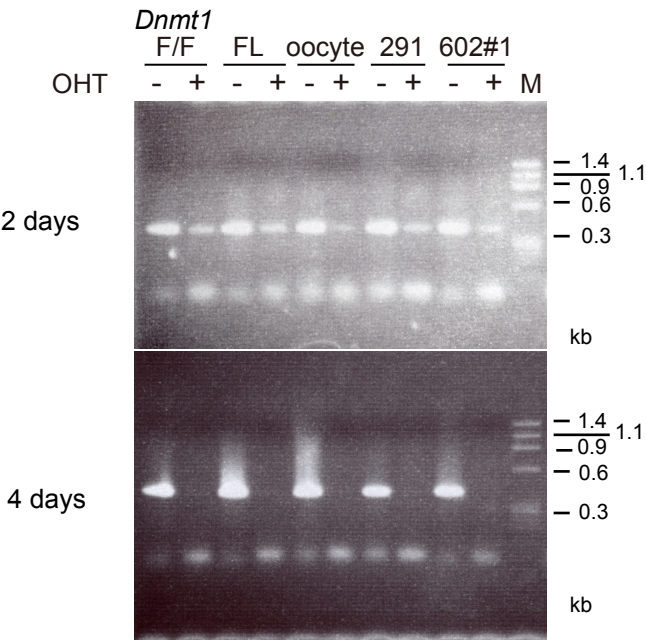

Supplement: S2 Fig — (A) Schematic illustration of the loxP inserted sites of the Dnmt1 gene, and the positions of the primers a and b (arrows). The numbers in boxes are exon numbers. To determine Dnmt1 deletion before and after OHT treatment, primers a and b, as follows were used. The amplification reaction comprised a cycle of denaturation at 94°C 2 min, and then 30 cycles of denaturation at 94°C 30 sec, annealing at 60°C for 30 sec, and extension at 72°C 1 min. Primer a: 5’-GTAAGTCTGTCCTTTTTCCCAGTTT-3’, and Primer b: 5’- AAACCAGTATGTCTCGTGTCCTTAC-3’. Successful deletion of the endogenous Dnmt1 diminishes amplification of the 351 bp + loxP (34 bp) size fragment with the (a + b) primer set. (B) PCR amplification of the endogenous Dnmt1 gene of Dnmt1-F/F cells, and cells ectopically expressing full-length Dnmt1 (FL), oocyte-type Dnmt1 (oocyte), Dnmt1(291–1620) (291), and Dnmt1(602–1620) (602#1), before (-) and after (+) OHT treatment. PCR amplifications were performed after two (upper panel) and four days (lower panel) treatment with OHT. Four days, not two days, treatment of the ESC with OHT completely deleted the endogenous Dnmt1 gene. Molecular size markers (M) are indicated at the right side of the gels. (PDF) [file pone.0137509.s002.pdf]
