# Supplementary material for: Dual Functions of the RFTS Domain of Dnmt1 in Replication-Coupled DNA Methylation and in Protection of the Genome from Aberrant Methylation
Source: PLoS One. 2015 Sep 18;10(9):e0137509. doi: 10.1371/journal.pone.0137509 (PMC4575159; doi:10.1371/journal.pone.0137509)
Supplement: S1 File — (DOCX) [file pone.0137509.s003.docx]

**Supporting Information**

**S1 File. Western blotting**

The isolated clones were solubilized with 1% SDS, electrophoresed in a 7.5% polyacrylamide gel, and then electrophoretically transferred to a nitrocellulose membrane (Pall). The membrane was blocked with 5% (w/v) skimmed milk in Dulbecco’s phosphate-buffered saline (PBS), and then incubated with the first antibodies in an antibody buffer comprising 1% (w/v) bovine serum albumin and 0.1% Triton X-100 in PBS. Anti-mouse Dnmt1 rabbit antibodies [1], anti-mouse Uhrf1 rat monoclonal antibody clone Th-10a (MBL, Japan) [2], anti-myc monoclonal antibody clone 4A6 (Millipore), anti-tubulin mouse monoclonal antibody clone DM1A (Sigma Aldrich), and anti-mouse Dnmt3a rabbit antibodies [3] were used as the first antibody, with incubation overnight at 4°C. After the incubation with the first antibodies, the membrane was washed and then incubated with antibodies conjugated with alkaline phosphatase, and visualized with nitroblue tetrazorium and 5-bromo-4-chloro-3-indolyl-phosphate [4].

1. Takagi H, Tajima S, Asano A. Overexpression of DNA methyltransferase in myoblast cells accelerates myotube formation. Eur J Biochem. 1995; 231: 282-291.

2. Muto M, Utsuyama M, Horiguchi T, Kubo E, Sado T, Hirokawa K. The characterization of the monoclonal antibody Th-10a, specific for a nuclear protein appearing in the S phase of the cell cycle in normal thymocytes and its unregulated expression in lymphoma cell lines. Cell Prolif. 1995; 28: 645-657.

3. Sakai Y, Suetake I, Shinozaki F, Yamashina S, Tajima S. (2004) Co-expression of *de novo* DNA methyltransferase Dnmt3a2 and Dnmt3L in gonocytes of mouse embryos. Gene Expr Patterns. 2004; 5: 231-237.

4. Harland RM. *In situ* hybridization: an improved whole-mount method for *Xenopus* embryos. Methods Cell Biol. 1991; 36: 685-695.
